# Case Report: Unmasking the role of rem sleep in modulating non-convulsive status epilepticus in ring chromosome 20 syndrome: a genetic disorder of sleep architecture?

**DOI:** 10.3389/fgene.2025.1626457

**Published:** 2025-08-13

**Authors:** Filippo Mandato, Maria Teresa Di Claudio, Umberto Costantino, Francesca Rovito, Orazio Palumbo, Pietro Palumbo, Angela La Neve, Maura Pugliatti, M. Castori, Massimo Carella, Giuseppe d’Orsi

**Affiliations:** ^1^ Neurology Unit, Epilepsy Center, IRCCS Casa Sollievo della Sofferenza, San Giovanni Rotondo, Italy; ^2^ Unit of Clinical Neurology, Department of Neuroscience and Rehabilitation, University of Ferrara, Ferrara, Italy; ^3^ UOC Genetica Medica, Fondazione IRCCS Casa Sollievo della Sofferenza, San Giovanni Rotondo, Italy; ^4^ Epilepsy Center, UOC Neurofisiopatologia, Bari, Italy

**Keywords:** ring chromosome 20 syndrome, non-convulsive status epilepticus, REM sleep, melatonin, EEG

## Abstract

Ring chromosome 20 syndrome (r (20)) is a rare genetic disorder characterized by drug-resistant epilepsy, cognitive impairment, and behavioral changes, often manifesting with non-convulsive status epilepticus (NCSE). We report a unique case of a 38-year-old woman with r (20) and recurrent NCSE, demonstrating a novel and striking electro-clinical correlation. Continuous video-EEG monitoring revealed distinct, alternating electro-clinical phases, with NCSE manifesting as continuous spike-wave during sleep (CSWS)-like patterns. Notably, the onset of REM sleep was consistently associated with a near-complete resolution of epileptiform abnormalities, followed by seizure-free awakening from REM. Intriguingly, the introduction of melatonin (4 mg/day) appeared to facilitate the attainment of REM sleep and was associated with a gradual reduction in NCSE frequency. This observation highlights the potential critical role of the neurophysiological state of REM sleep – characterized by cholinergic predominance and active GABAergic inhibition – in modulating and potentially suppressing the aberrant cortical excitability underlying NCSE in r (20). We hypothesize that the disrupted sleep architecture in r (20) may contribute to NCSE vulnerability, and that enhancing REM sleep dynamics could counteract this predisposition. The observed benefit of melatonin, potentially acting on MT1 receptors, warrants further investigation into targeted interventions aimed at normalizing sleep architecture, particularly REM sleep, as a novel therapeutic strategy for managing drug-resistant epilepsy and NCSE in r (20). This case underscores the importance of REM sleep in the context of epilepsy in r (20) and calls for future studies to elucidate the underlying mechanisms and confirm these findings in larger cohorts.

## Introduction

Ring chromosome 20 syndrome (r (20)) is a rare, genetically and clinically defined syndrome resulting from a non-supernumerary ring chromosome 20 replacing a normal chromosome 20, most commonly occurring in a mosaic state (Peron et al., 2020). Its phenotype is characterized by drug-resistant epilepsy with a typical electroencephalography (EEG) pattern, cognitive impairment manifesting after seizure onset in children with previously normal development, and behavioral changes. A triad of electroclinical features has been described: drug-resistant frontal lobe seizures and recurrent non-convulsive status epilepticus (NCSE). These are often accompanied by characteristic EEG features such as brief frontal epileptic discharges, long-lasting high-voltage slow waves, bilateral frontal spikes, and trains of theta waves in frontotemporal areas, exhibiting a specificity of 63.3% and a sensitivity of approximately 100% (Gago-Veiga et al., 2018). As previously reported, the optimal anti-seizure medications (ASMs) appear to be the combination of valproic acid and lamotrigine; however, seizures remained drug-resistant in the majority of patients (Vignoli et al., 2016). The sleep architecture in this syndrome remains poorly understood. While some studies have investigated the disruption of sleep architecture and alterations in NREM/REM cycles in r (20) (Zambrelli et al., 2013), to our knowledge, no studies have specifically focused on REM sleep phases and their relationship with NCSE in this syndrome. We present a case of r (20) and epilepsy in which the cessation of NCSE coincided with the attainment of REM sleep, followed by awakening in a fully oriented state. Intriguingly, the administration of melatonin appeared to facilitate the achievement of REM sleep. Consequently, interventions aimed at improving REM sleep duration and regulating sleep phases appeared to positively impact the patient’s quality of life and social interaction, with an apparent reduction in both overt seizures and NCSE burden. These novel observations highlight the potential critical role of REM sleep in modulating epileptic activity in r (20) and warrant further investigation.

## Case report

**FIGURE 1:**
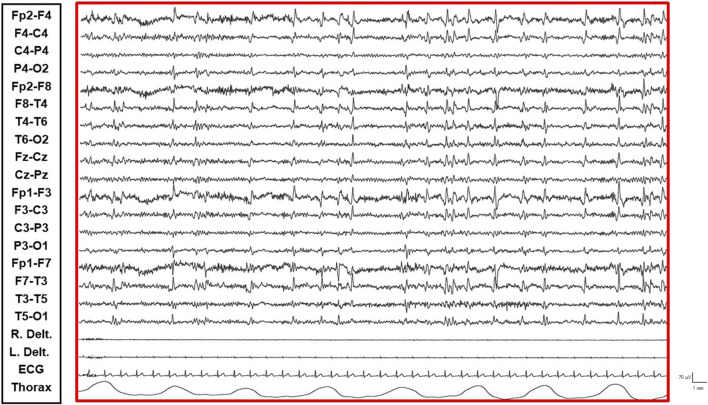
Restful Phase: Patient exhibits vigilance fluctuations. EEG shows an increased number of diffuse epileptic abnormalities.

**FIGURE 2:**
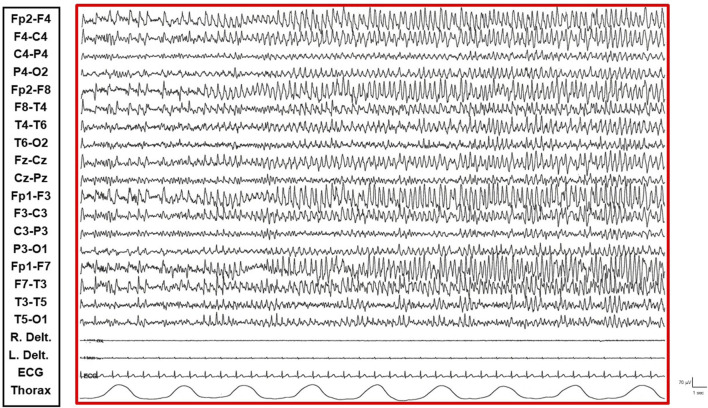
Transition Phase: Patient presents with decreased vigilance and somnolence. EEG shows repetitive and diffuse epileptic abnormalities tending towards rhythmic and recruiting patterns.

**FIGURE 3:**
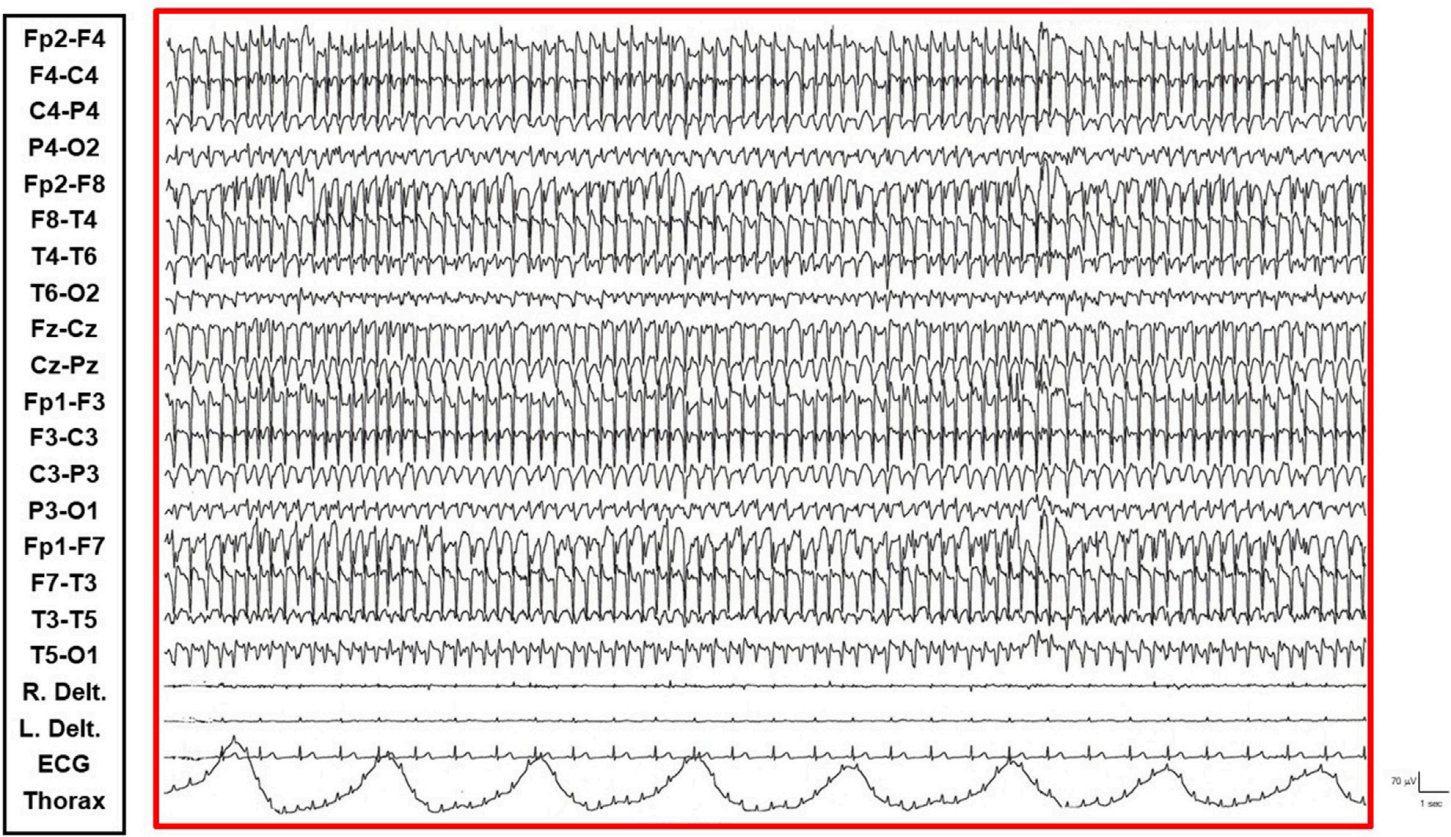
NCSE Phase: Continuous, diffuse, and symmetrical epileptic abnormalities consistent with CSWS are observed.

**FIGURE 4:**
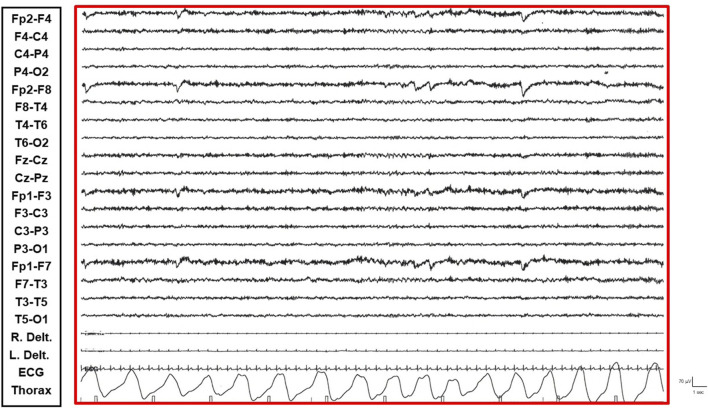
REM Sleep Phase: Epileptic abnormalities have resolved.

The patient is a 38-year-old, right-handed woman born to non-consanguineous parents with no familial history of epilepsy. Her prenatal and perinatal periods were unremarkable, with normal developmental milestones and no reported dysmorphic features. At 4.5 years of age, she was diagnosed with drug-resistant epilepsy. Later, at the age of 12 years, she underwent karyotype analysis using lymphocytes collected from peripheral blood resulting a mosaic for the ring chromosome 20. The result was 46,XX [44]/46,XX,r (20) (p13q13)[56]. In order to rule out the potential presence of pathogenic copy number variations not identifiable by classical cytogenetics, SNP-array analysis was performed as previously described (Palumbo et al., 2020) and yielded negative results. Clinically, her seizures were characterized by daily episodes of cognitive slowing and prominent visual hallucinations, accompanied by mild cognitive impairment. Over the following years, she underwent trials of various anti-seizure medication (ASM) polytherapies, including carbamazepine prolonged-release (CBZ-PR), lamotrigine (LTG), valproic acid (VPA), clobazam (CLB), phenytoin (PHT), ethosuximide (ESM), vigabatrin (VGB), phenobarbital (PB), felbamate (FBM), topiramate (TPM), and levetiracetam (LEV), without achieving complete seizure control. A regimen of VPA Chrono 1250 mg/day, LTG 400 mg/day, and CLB 10 mg/day resulted in a relatively favorable quality of life, allowing for adequate autonomy in daily activities. Notably, during the subsequent years, despite persistent multi-monthly absence seizures and multi-annual NCSE episodes, she established a partnership, obtained a university degree, and authored a published book. Multiple EEGs performed over the years revealed diffuse slow and epileptiform abnormalities, occasionally bilaterally asynchronous with temporal predominance. These abnormalities were exacerbated by fluctuations in vigilance and by sleep. Furthermore, a loss of recognizable NREM sleep stages was observed, characterized by high-amplitude delta sequences with sharply contoured or notched morphology, prevalent over frontal regions. At the age of 37, cannabidiol (CBD) was added to her medication regimen, with the dosage titrated up to 6 mL per day. The initial 20 days of this combination therapy were associated with a progressive improvement in her clinical status, including improved nocturnal sleep duration and quality, and apparent complete seizure cessation – an unprecedented improvement in her history. However, subsequent blood analysis revealed elevated levels of liver transaminases (aspartate transaminase 141 U/L [reference range not provided], alanine transaminase 230 U/L [reference range not provided], gamma-glutamyltransferase 112 U/L [reference range not provided]). Consequently, her neurologist decided to gradually reduce the VPA dosage (to 1,000 mg/day) and discontinue CBD entirely. This therapeutic modification precipitated recurrent, prolonged, drug-resistant, and super-refractory non-convulsive status epilepticus (NCSE) episodes, necessitating multiple hospitalizations. She exhibited no response to benzodiazepines (BDZs) and even failed burst suppression therapy in the Intensive Care Unit. Various ASMs were attempted, including lacosamide (LCM), PHT, CLB, LEV, midazolam (MDZ), burst suppression with propofol for several days, and stiripentol. Following these hospitalizations, she was discharged on a regimen of VPA chrono 1500 mg/day, LTG 150 mg/day, stiripentol 1,500 mg/day, and LEV 3000 mg/day. With this medication regimen, she was referred to our center. Upon presentation to our Emergency Room, she exhibited multiple episodes of cognitive slowing, delayed responses to simple questions and commands, and an inability to perform complex tasks, with no response to BDZs. This clinical picture was associated with an EEG pattern consistent with NCSE, characterized by repetitive, rhythmic, and recruiting epileptic discharges, diffusely distributed but with a frontal bilateral predominance. The initial management strategy involved 72-h video-EEG monitoring to guide further therapeutic modifications. Electro-clinical evaluation during her hospitalization identified five distinct phases alternating throughout the daytime, each characterized by specific electro-clinical patterns:

• Active Phase: During the active phase, the patient was fully awake, oriented, and capable of performing activities of daily living and complex tasks (e.g., playing cards, using her phone or laptop). The EEG pattern during this phase showed sporadic and isolated epileptic abnormalities, such as spikes and spike-wave complexes, sometimes with anterior predominance (Supplementary Figure S5).
• Restful Phase: When unstimulated and alone in her room, watching television or videos, the patient exhibited occasional vigilance fluctuations. The EEG showed a gradual increase in the number of diffuse epileptic abnormalities, enhanced by eye closure. Awakening from this state by external stimuli (e.g., loud noises, movements) was associated with a return of the EEG to the active vigil state pattern (Figure 1).
• Transition Phase: Following prolonged periods of the restful vigil state, the patient began to experience decreased vigilance, somnolence, and dizziness. The EEG pattern showed repetitive and diffuse epileptic abnormalities that tended to become rhythmic and recruiting. During these episodes, the patient appeared dizzy, less reactive, and occasionally experienced emotional episodes of fear (Figure 2).
• Non-Convulsive Epileptic State (NCSE) Phase: Predominantly occurring during sleep and replacing NREM phases, this state was characterized by a continuous EEG pattern of rhythmic, recruiting, diffuse, and symmetrical epileptic abnormalities consistent with continuous spike-wave during sleep (CSWS) (Figure 3).
• REM Sleep Phase: The onset of eye movement artifacts was followed by a sudden shift in the EEG pattern to a rapid, symmetrical, desynchronized activity with reduced amplitude. Epileptic abnormalities almost completely resolved, persisting sporadically in the bilateral frontal derivations. Notably, the patient regained an active vigil state free from epileptic abnormalities only upon awakening from REM sleep phases. Conversely, awakening from NCSE resulted in the persistence of the epileptic state even while awake (Figure 4).

The introduction of melatonin (4 mg/day) gradually facilitated the attainment of REM sleep, with a concurrent gradual reduction in NCSE frequency (Supplementary Figure S6). However, at a 6-month follow-up, the electro-clinical presentation had returned to a pattern comparable to previous years.

## Discussion

This case report describes a 38-year-old woman with drug-resistant epilepsy secondary to r (20), who experienced recurrent NCSE. Notably, our detailed video-EEG monitoring revealed that the disappearance of NCSE coincided with the attainment of REM sleep, followed by the patient’s return to an active vigil state and orientation upon awakening from REM sleep, free from epileptic activity. This observation highlights the potential critical role of REM sleep in modulating and resolving NCSE in this specific case, with melatonin and possibly CBD likely playing a role in facilitating REM sleep and, consequently, NCSE control. Zambrelli and colleagues (2013) observed a loss of recognizable NREM sleep stages, lacking the characteristic hallmarks of normal sleep such as K-complexes and spindles, and a progressive, potentially age-dependent, deterioration of overall sleep structure in individuals with r (20). The distinctive sleep pattern they described, characterized by high-amplitude delta sequences with sharply contoured or notched morphology, prevalent over frontal regions, bears resemblance to the findings in our patient, suggesting a potentially conserved sleep pattern in r (20). The underlying mechanisms for this altered sleep architecture in r (20) may involve dysregulation of the basal ganglia, with a significant role for dopamine uptake in the caudate and putamen (Lee et al., 2007). This disruption could impact the intricate balance of neurotransmitters and neural circuits crucial for the generation and maintenance of normal sleep stages. Specifically, altered dopaminergic signaling could interfere with the transitions between sleep states and the stability of NREM and REM sleep. The observed predominance of NCSE during sleep, particularly replacing NREM phases, further underscores the vulnerability of sleep-related neural networks in r (20) to pathological synchronization. Notably, the relationship between REM sleep and awakening in r (20) has received limited attention, warranting further investigation in a larger cohort of patients. A study by Augustijn et al. (2001) reported the disappearance of epileptiform activity during REM sleep, suggesting a potential reduction of epileptic activity during this phase, which could contribute to seizure-free awakening, consistent with our observation. The neurophysiological milieu of REM sleep, characterized by a relative cholinergic predominance, active neuronal inhibition mediated by GABAergic mechanisms, and a functional decoupling from external sensory input, may create an environment less conducive to the generation and propagation of the sustained, synchronous neuronal firing characteristic of NCSE. The reduction in slow-wave activity and the increase in faster frequencies during REM sleep could also contribute to the disruption of the pathological slow-wave oscillations seen in CSWS. In our patient, we hypothesize that cannabidiol (CBD) may have contributed to the temporary resolution of NCSE during the 20 days of combined therapy. Several studies have indicated the efficacy of CBD in reducing seizure frequency in various drug-resistant epilepsies (Klotz et al., 2021; Ferrera et al., 2023). While its direct effects on sleep phases are not well-established, potential mechanisms could involve modulation of endocannabinoid signaling, which is known to influence both sleep and seizure susceptibility. The reported increase in slow-wave sleep (SWS) and REM sleep with CBD in insomnia (Wang et al., 2025) suggests a possible, albeit indirect, influence on REM sleep in our patient. Given the hepatic transaminase elevation with CBD, we opted for melatonin (4 mg per day) as an alternative. The observed reduction in NCSE frequency following melatonin introduction aligns with findings that melatonin receptor 1 (MT1 receptor) agonists can promote REM sleep duration (López-Canul et al., 2024). Activation of MT1 receptors, located in key sleep-regulating areas such as the locus coeruleus and lateral hypothalamus (Gobbi and Comai, 2019), may enhance the physiological processes underlying REM sleep, potentially counteracting the pathological synchronization seen in NCSE. The observed increase in NREM sleep and decrease in REM sleep in MT1 knockout mice further supports the role of this receptor in REM sleep regulation. Thus, melatonin’s beneficial effect on NCSE in our patient may be mediated through the restoration or enhancement of REM sleep, thereby leveraging the intrinsic anti-epileptic properties of this sleep stage. In conclusion, this case suggests a possible correlation between the disruption of sleep phases, specifically a reduction in REM sleep duration and the substitution of NREM phases with CSWS, and the patient’s return to an active vigil state and orientation upon awakening from REM sleep. Our case suggests that pharmacological interventions targeting sleep regulation, particularly REM sleep enhancement, could potentially mitigate daytime NCSE and improve the quality of life for individuals with Ring20 Syndrome. In our patient, both melatonin and possibly CBD showed potential benefits; furthermore, this case underscores the potential importance of REM sleep in the context of NCSE in r (20). We recommend that future studies dedicate further investigation to the role of REM sleep in this condition to better characterize its involvement in seizure control and inform potential therapeutic strategies, including targeted interventions aimed at normalizing sleep architecture and enhancing REM sleep. Notably, the observed resolution of NCSE coinciding with REM sleep in this patient with r (20) syndrome highlights an unexpected and potentially crucial neurophysiological interaction, offering a window into the underlying aberrant neurobiology.

## Data Availability

The raw data supporting the conclusions of this article will be made available by the authors, without undue reservation.
