# Supplementary figures and images for: Case Report: Unmasking the role of rem sleep in modulating non-convulsive status epilepticus in ring chromosome 20 syndrome: a genetic disorder of sleep architecture?
Source: Front Genet. 2025 Aug 13;16:1626457. doi: 10.3389/fgene.2025.1626457 (PMC12380844; doi:10.3389/fgene.2025.1626457)

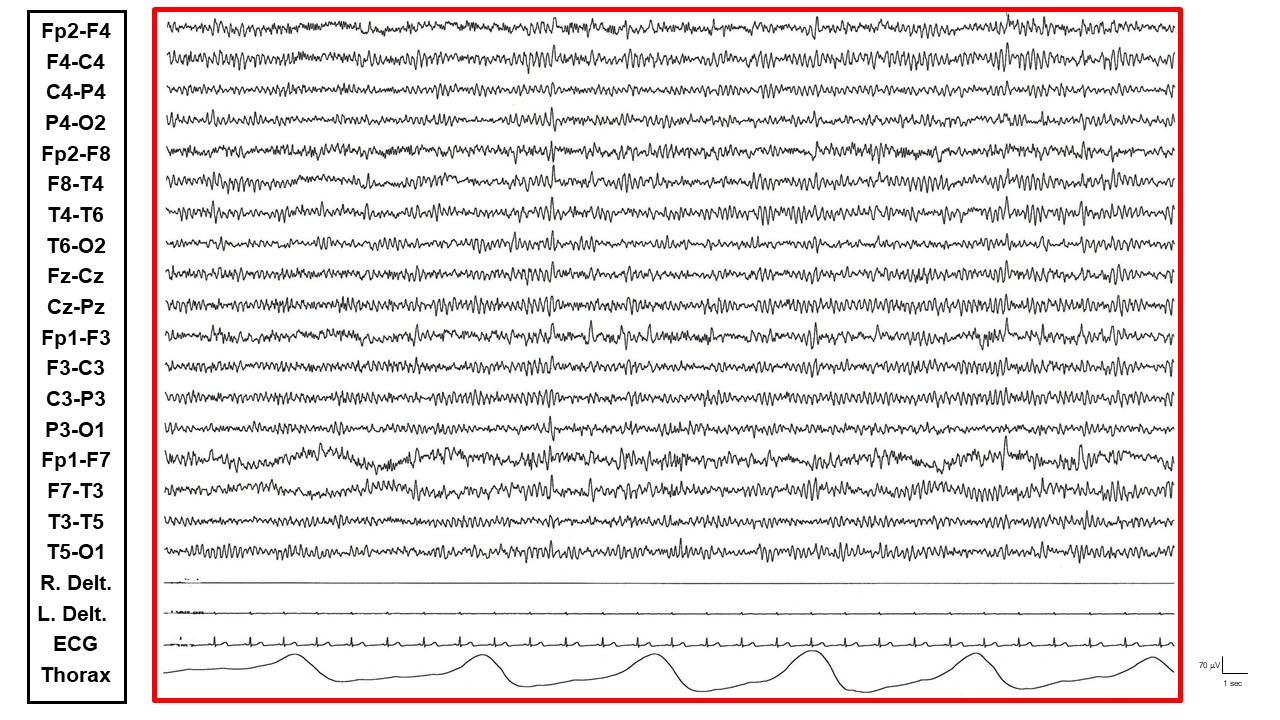

Supplement: Supplementary file 1 [file Image1.jpeg]

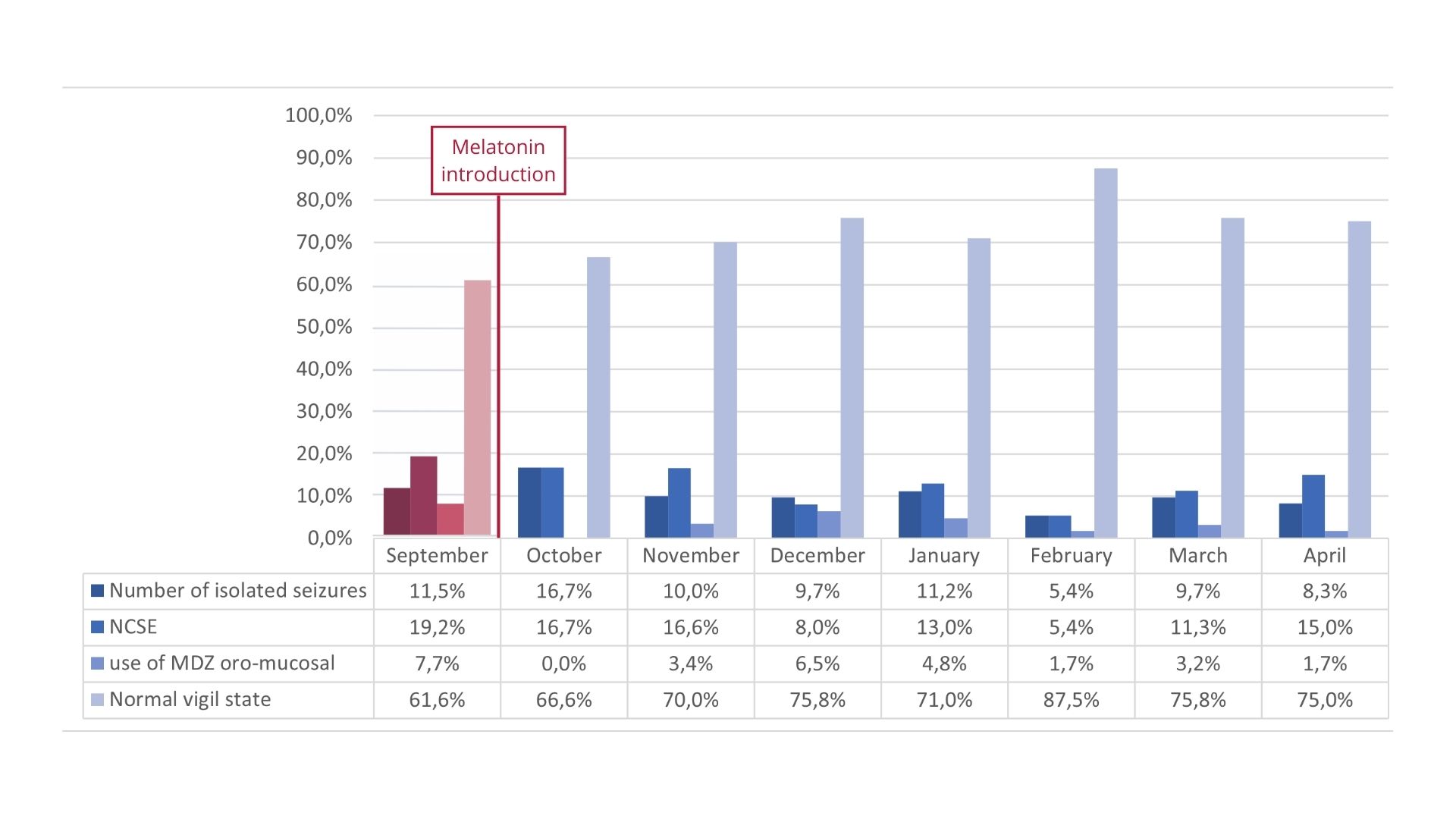

Supplement: Supplementary file 2 [file Image2.jpeg]
